# Hypophosphatasia: Results of a Country-Wide Selective Screening Program Using NGS Technology as a First-Tier Test

**DOI:** 10.3390/ijms27157008

**Published:** 2026-08-04

**Authors:** Aleksander A. Pushkov, Ilya S. Zhanin, Daria A. Chudakova, Anastasia A. Rusakova, Dmitry S. Demianov, Aleksander V. Pakhomov, Valeriya B. Koroleva, Yuliya S. Koshevaya, Anastasia S. Burlachenko, Yury A. Eismont, Oleg S. Glotov, Andrey P. Fisenko, Kirill V. Savostyanov

**Affiliations:** 1 National Medical Research Center of Children’s Health, Ministry of Health of the Russian Federation, 119991 Moscow, Russia; 2 Center for Precision Genetic Technologies for Medicine, Engelhardt Institute of Molecular Biology, Russian Academy of Sciences, 119991 Moscow, Russia; 3 Center for Genomic Technologies «Cerbalab», 199106 Saint-Petersburg, Russiaolglotov@mail.ru (O.S.G.); 4 Federal State Budgetary Institution «Federal Scientific and Clinical Center for Infectious Diseases of the Federal Medical and Biological Agency», 197022 Saint-Petersburg, Russia; 5 State Budgetary Healthcare Institution of the City of Moscow «Moscow Scientific and Practical Center of Laboratory Research of the Moscow Healthcare Department», 115580 Moscow, Russia

**Keywords:** hypophosphatasia, HPP, tissue-nonspecific alkaline phosphatase, *ALPL* gene

## Abstract

Hypophosphatasia (HPP) is a rare hereditary metabolic disorder caused by nucleotide variants (NVs) in the *ALPL* gene, leading to a deficiency of tissue-nonspecific alkaline phosphatase (TNSALP). HPP has a variable clinical presentation (such as impaired mineralization of bones and teeth, respiratory dysfunctions, muscle weakness, muscle and bone pain, growth deficiency, nephrocalcinosis, seizures, encephalopathy, intracranial hypertension, and intellectual disability) overlapping with other disorders which often delays a correct diagnosis. In this study, we present the results of a two-center (Moscow and Saint Petersburg) selective screening program conducted in the period 2022–2026 in Russia, based on next-generation sequencing (NGS) of 4912 patients with suspected HPP. Each center used its own NGS gene panel. In Moscow, causal nucleotide variants in *ALPL* were found in 9.45% of patients, and variants in other genes from the panel were identified in 9.77% of patients. The selection criteria for the screening evolved during the study, resulting in a higher diagnostic yield. In Saint Petersburg, causal variants in *ALPL* were found in 3.5% of patients. The most frequent *ALPL* variant was c.571G>A (28%), which is consistent with its founder effect in Northern Europe. In the differential diagnosis gene panel, pathogenic or likely pathogenic variants were most commonly found in the *COL1A2*, *COL1A1*, and *CASR* genes, highlighting the phenotypic overlap of HPP with osteogenesis imperfecta and related disorders, and underscoring the value of targeted NGS for resolving diagnostic uncertainty in HPP. Overall, this study provides the most comprehensive data on the genetics of HPP in the Russian population to date.

## 1. Introduction

Hypophosphatasia (HPP) is a rare inherited metabolic disorder caused by deficiency or dysfunction of the enzyme tissue-nonspecific alkaline phosphatase (TNSALP or TNAP; UniProt: P09242), resulting from the presence of causal nucleotide variants (NVs) in the *ALPL* gene (MIM# 171760, also known as *HOPS*, *TNALP*, *TNAP*, *TNSALP*) [1]. HPP can be inherited either in a biallelic autosomal recessive (AR) or monoallelic autosomal dominant (AD) manner [2]. TNSALP belongs to the alkaline phosphatase family of enzymes (ALPs; orthophosphoric monoester phosphohydrolases (alkaline optimum), EC 3.1.3.1), which in humans also includes three tissue-specific ALPs: placental (encoded by the *ALPP* gene, GeneID: 250), fetal (encoded by the *ALPG* gene, GeneID: 251), and intestinal (encoded by the *ALPI* gene, GeneID: 248). *ALPP*, *ALPG*, *ALPI* and *ALPL* are homologous genes clustered together on chromosome 2 and showing a high degree of sequence identity (~90%), whereas *ALPL* is located separately and shares only ~50% sequence identity with these tissue-specific ALPs genes [3]. It is located on chromosome 1p36.1-p34 [4] and consists of 12 exons, including 11 protein-coding exons.

More than 450 nucleotide variants have been described in the *ALPL* gene, which can result in various clinical manifestations of HPP. A constantly updated database with information on these variants has been created (https://alplmutationdatabase.jku.at/the Johannes Kepler University ALPL Gene Variant Database at the Johannes Kepler University in Linz, Austria, accessed on 30 June 2026).

TNSALP is a transmembrane N-glycosylated glycoprotein, anchored in the cell membrane by glycosylphosphatidylinositol anchor, facing the extracellular space. There are three isoforms of TNSALP, mainly expressed in the liver, bone tissue and kidneys (hence it is also referred to as a “liver–bone–kidney-type alkaline phosphatase”) and differing in glycosylation pattern [5]. There are five putative *N*-glycosylation sites at asparagine (N) residues 140, 230, 271, 303, 430) in TNSALP [6]. The earlier studies indicated that TNSALP is an obligatory homodimer; recently, it has been demonstrated that such a dimer undergoes subsequent tetramerization and octamerization [7]. It is predominately localized on cell surface, specifically cell plasma membrane, or can be secreted into extracellular space and circulation; in some cells (brown adipocytes) it is found anchored to mitochondrial membrane [8].

TNSALP circulating in the bloodstream accounts for more than 50% of the total ALP activity in blood. Hence, ALP activity in blood can be used as a reliable proxy of TNSALP activity in tissues [9].

TNSALP is involved in a multitude of pathways and governs several biological processes, depending on and regulated by its substrates. The main physiological substrates of TNSALP are inorganic pyrophosphate, pyridoxal-5′phosphate (the active form of vitamin B6 and a cofactor of many enzymes, including those involved in neurotransmitter synthesis), and phosphoethanolamine [1]. Several other substrates of TNSALP have been described, for example, phosphorylated osteopontin, phosphocreatine (which is hydrolyzed by mitochondria-localized TNSALP in brown fat adipocytes) [8], and others.

TNSALP deficiency leads to multiple and systemic pathological alterations. HPP may manifest as an impaired mineralization of bones and teeth, accompanied by systemic complications including respiratory dysfunctions, muscle weakness, muscle and bone pain, growth deficiency, nephrocalcinosis, and a spectrum of neurological symptoms (seizures, encephalopathy, intracranial hypertension, intellectual disability, etc.).

Perinatal severe/lethal HPP, as the name suggests, is the most severe, life-threatening form. Other forms vary in severity, clinical signs and age of onset (infantile < 6 months; childhood > 6 months to <18 years; adult > 18 years). Perinatal benign HPP is an extremely rare form. It is characterized by prenatal pathological features of skeletal formation, which eventually resolve, and later the patient develops a moderate form of HPP. Mild forms of HPP often remain undiagnosed.

Hitherto, HPP therapy is based on enzyme replacement therapy (ERT) with recombinant TNSALP, Asfotase Alfa (Strensiq) [10]. ERT is mainly used for the treatment of HPP in infants and young children, showing drastic improvements in survival, skeletal mineralization, motor and cognitive functions [10]. At the same time, adults with HPP may also benefit from ERT. For example, it has been demonstrated that ERT leads to significant improvements in mobility, physical activity, and quality of life of adult patients with HPP [11]. Thus, timely diagnosis is undoubtedly significant for some patients, and of critical importance for many others.

HPP is a rare disease. A study evaluating the period 2000–2009 determined the incidence of severe forms of HPP in various parts of Europe ranging from 1:297,000 to 1:1,358,000 [12]. Moderate forms of HPP are much more common. Their estimated frequency in Europe is ~1:2430 [12]. Due to the rarity of this disease and a wide range of symptoms that overlap with other diseases, it usually takes a long time to establish the correct diagnosis in patients with HPP. Some patients get diagnosis of HPP as adults, despite disease manifestation in childhood. Significant diagnostic delay, a long “Diagnostic Odyssey” of patients with HPP, is well-documented [13]. Notably, low ALP levels might be observed in case of many other conditions, namely celiac disease, Cushing syndrome, hypothyroidism, multiple myeloma, cardiac bypass surgery, Clofibrate therapy, osteogenesis imperfecta (OI) type 2, pernicious anemia, massive transfusion, milk-alkali syndrome, profound anemia, starvation, radioactive heavy metals, vitamin C deficiency, vitamin D intoxication, Wilson disease, and others [14].

There is no “gold standard” approach to diagnosing HPP. In 2023, the HPP International Working Group, based on an analysis of 24 publications from Asia, 42 from Europe, 24 from North America, 1 from South America, and 2 from several continents, summarized clinical manifestations of HPP in children. The presence of pathogenic or likely pathogenic NVs (P/LP NVs) in the *ALPL* gene in 41 articles (79%) was indicated as the most convincing sign suggesting HPP. Elevated levels of natural substrates of TNASP were indicated as a convincing sign of HPP in 37 articles (71%). Finally, early non-traumatic tooth loss was mentioned as a clinical sign of HPP in 32 articles (61%) [15]. In Russia, diagnosis of HPP is guided by clinical recommendations [16] subject to periodic revision, ensuring alignment with current scientific evidence and evolving diagnostic methods. Hitherto, sequencing of *ALPL* for establishing definitive diagnosis of HPP is not mandatory anywhere in the world but recommended [17]. Further, the differential diagnosis of HPP may rely on next-generation sequencing (NGS) of large gene panels or whole-genome sequencing [18].

Our current work aims to further advance the understanding of molecular and genetic features of HPP in the Russian population and develop approaches to differential diagnosis of this rare disease. In the current study, we developed a panel of 57 genes involved in various pathological conditions with clinical features of bone fragility and increased bone fracture risk, as well as others, overlapping with HPP, and in 2023–2026, we performed a targeted next-generation sequencing (NGS) of this panel in a cohort of 3121 pediatric and adult patients. We are publishing data from a two-center study. The second center, located in St. Petersburg, also used NGS as a first-line test, but the panel did not include differential diagnosis genes. Here, we present results of this study, including frequency of HPP in the study population (*n* = 4912 in total), spectrum of causative NVs in the *ALPL* gene, and NVs in other genes from the panel.

## 2. Results

### 2.1. Experiment Workflow

In 2023–2026 in the National Medical Research Center of Children Health, Moscow, Russian Federation, we performed a targeted NGS of a panel of 57 genes involved in pathological conditions with clinical features overlapping with HPP in a cohort of 3121 patients from various regions of Russian Federation (hereafter referred as the Moscow cohort). The sex ratio in the cohort was 47% male and 53% female; the median age was 12 years. There were 2523 patients under 18 years of age (81%) and 598 adults (19%).

In 2023–2025 another participant, the Center for Genomic Technologies (Cerbalab) Saint Petersburg, Russian Federation, also performed NGS in a cohort of 1791 patients with various symptoms suggesting HPP (hereafter referred to as the Saint Petersburg cohort). The sex ratio in the cohort was 42% male and 48% female; the median age was 9 years. There were 1612 patients under 18 years of age (90%) and 179 adults (10%).

### 2.2. NGS Analysis

**Figure 1:**
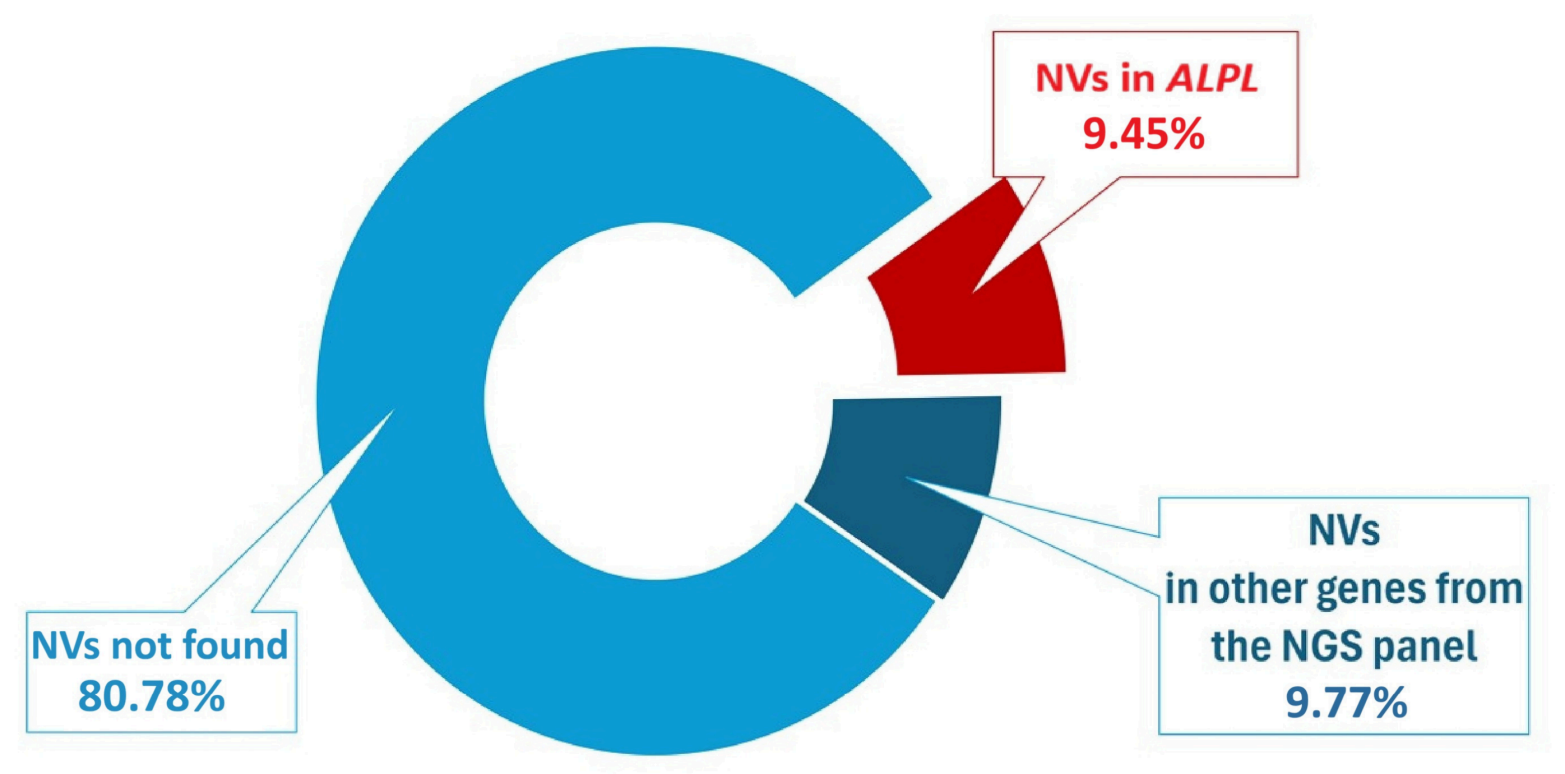
Distribution of genetic variants in the *ALPL* and other panel genes based on NGS data.

The NGS analysis of 3121 patients from the Moscow cohort revealed that 9.45% (*n* = 295) had NVs in the *ALPL* gene, 9.77% (*n* = 305) had NVs in other genes from the panel, and the remaining 80.78% (*n* = 2521) did not have NVs in all genes tested (Figure 1).

In total, 4912 patients (Moscow and Saint Petersburg cohorts) with suspected HPP were examined using the NGS method.

The study program was divided into three time periods, based on changes in patient selection criteria (see Section 4.1). A total of 3121 patients with suspected HPP were referred to Laboratory 1 (Moscow cohort) and genotyped. The total number of patients with causal *ALPL* gene variants identified in this program from September 2023 to March 2026 was 295 (9.5%). The detection rate varied by year depending on the adopted selection criteria. Thus, during the first period of the screening program (September 2023–February 2024), 306 patients were analyzed, and the detection rate was 3.3%. The second period (February 2024–August 2024) showed a detection rate of 5% (41/824). In the third period (August 2024–March 2026), the detection rate was 12.2% (244/1991).

From March 2022 to December 2025, Laboratory 2 (Saint Petersburg cohort) identified 64 patients with causal variants of the *ALPL* gene, which amounted to 3.5% (63/1791).

**Figure 2:**
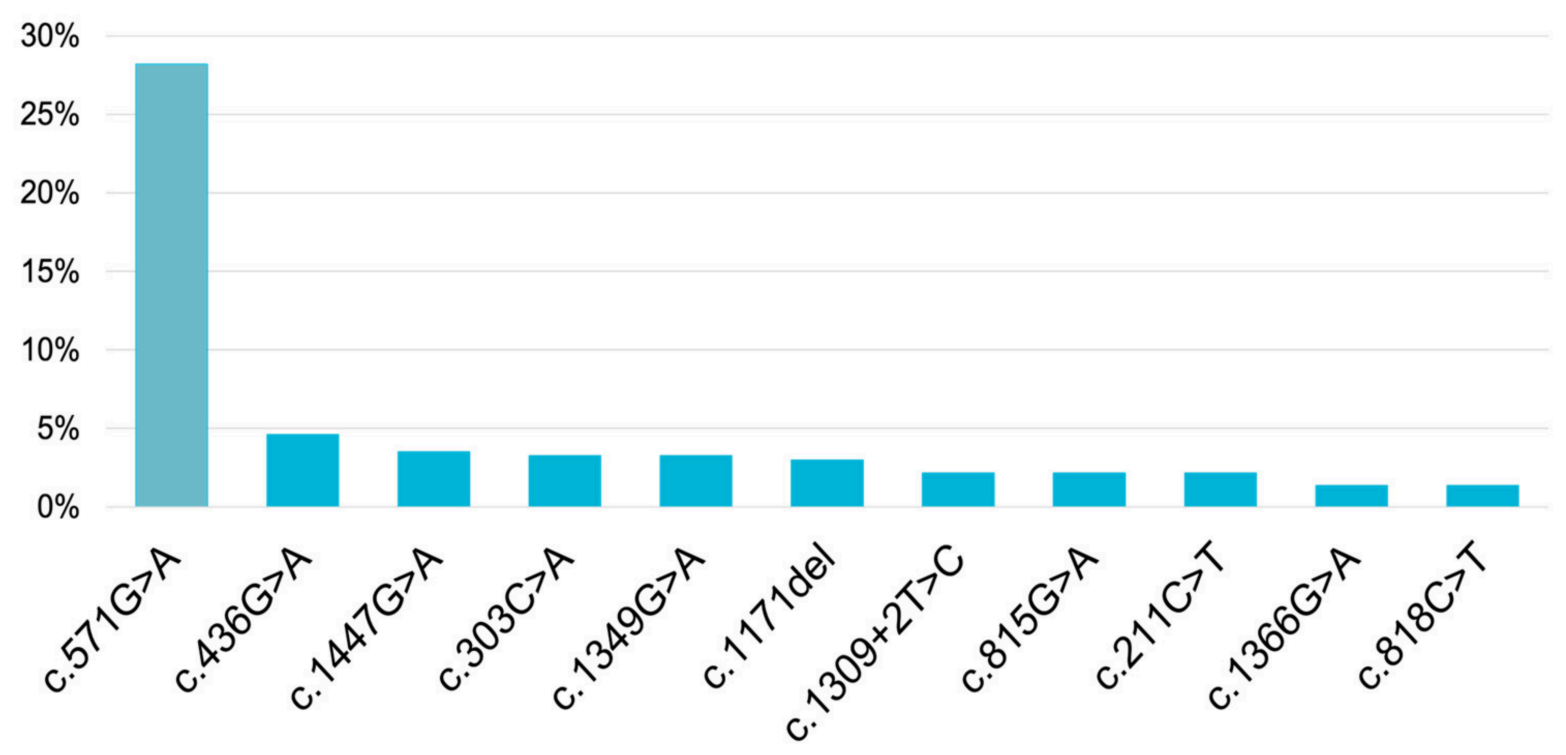
Frequencies of the most common causal nucleotide variants in *ALPL*.

Pathogenic variants, likely pathogenic variants, and variants of uncertain clinical significance in the *ALPL* gene were identified in total in 358 patients on 371 alleles. The most frequent variants were c.571G>A-104/371 (28.0%), c.436G>A-17/371 (4.6%), c.1447G>A-13/371 (3.5%), c.1349G>A and c.303C>A-12/371 (3.2%), c.1171del-11/371 (3.0%), c.1309 + 2T>C, c.815G>A, and c.211C>T-8/371 (2.2%), c.1366G>A and c.818C>T-5/371 (1.4%) (Figure 2).

In 10 patients, the variants were identified in a compound heterozygous state, whereas in three patients the variants c.1171del, c.571G>A, and c.1001G>A were detected in a homozygous state. A total of 123 unique variants were identified, among which 70 (56.9%) were classified as pathogenic, 37 (30.0%) as likely pathogenic, and 16 (13.0%) as variants of uncertain clinical significance.

**Figure 3:**
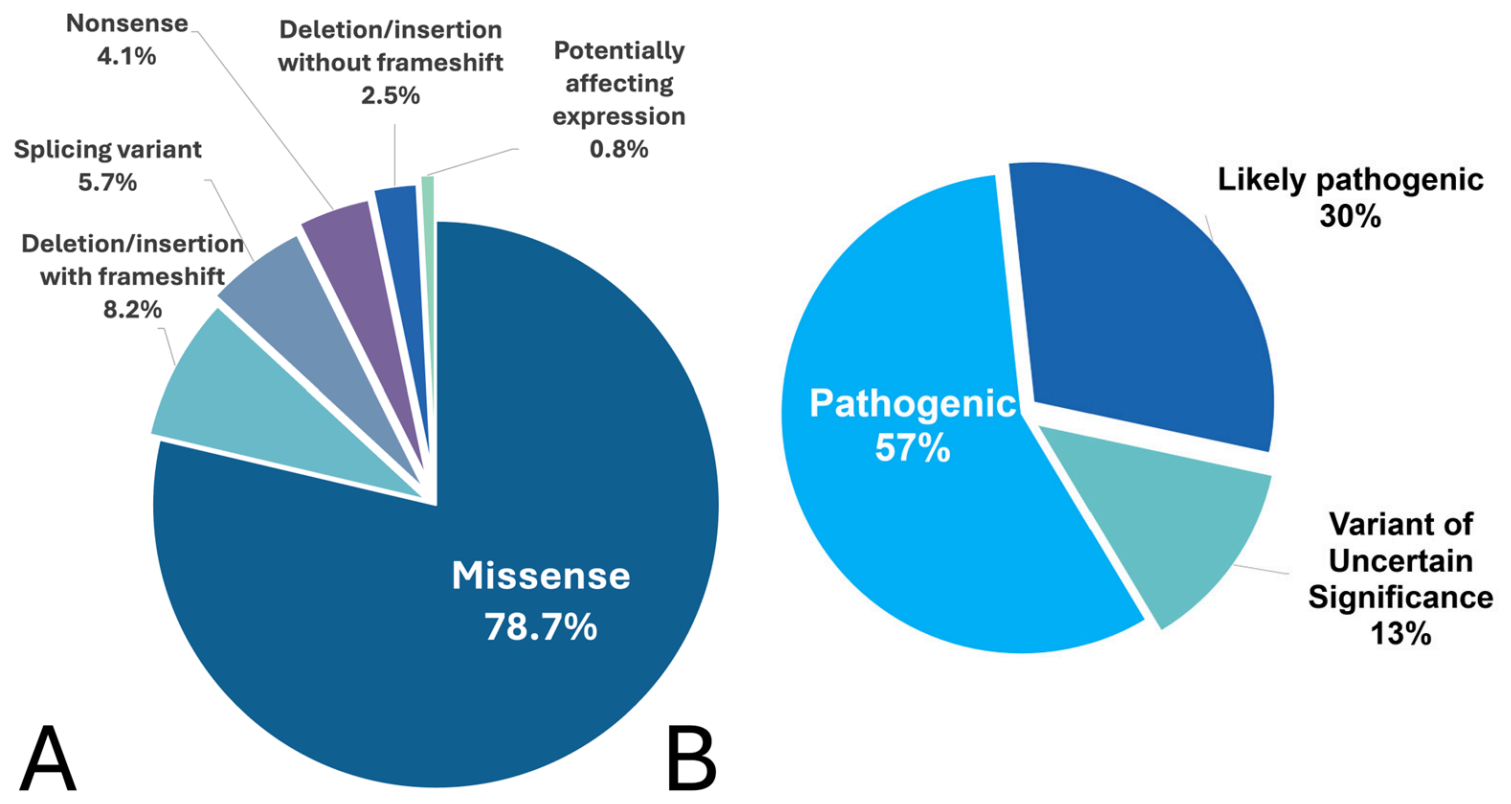
Characteristics of nucleotide variants found in *ALPL* gene. Types (**A**) and pathogenicity (**B**) of identified variants.

According to mutation type, the variants were distributed as follows: missense variants—96 (78.7%); frameshift deletions/insertions—10 (8.2%); intronic variants—7 (5.7%); nonsense variants—5 (4.1%); in-frame deletions/insertions—3 (2.5%); variants with a presumed effect on gene expression—1 (0.8%). Thus, the predominant type of alteration consisted of missense variants (Figure 3).

**Figure 4:**
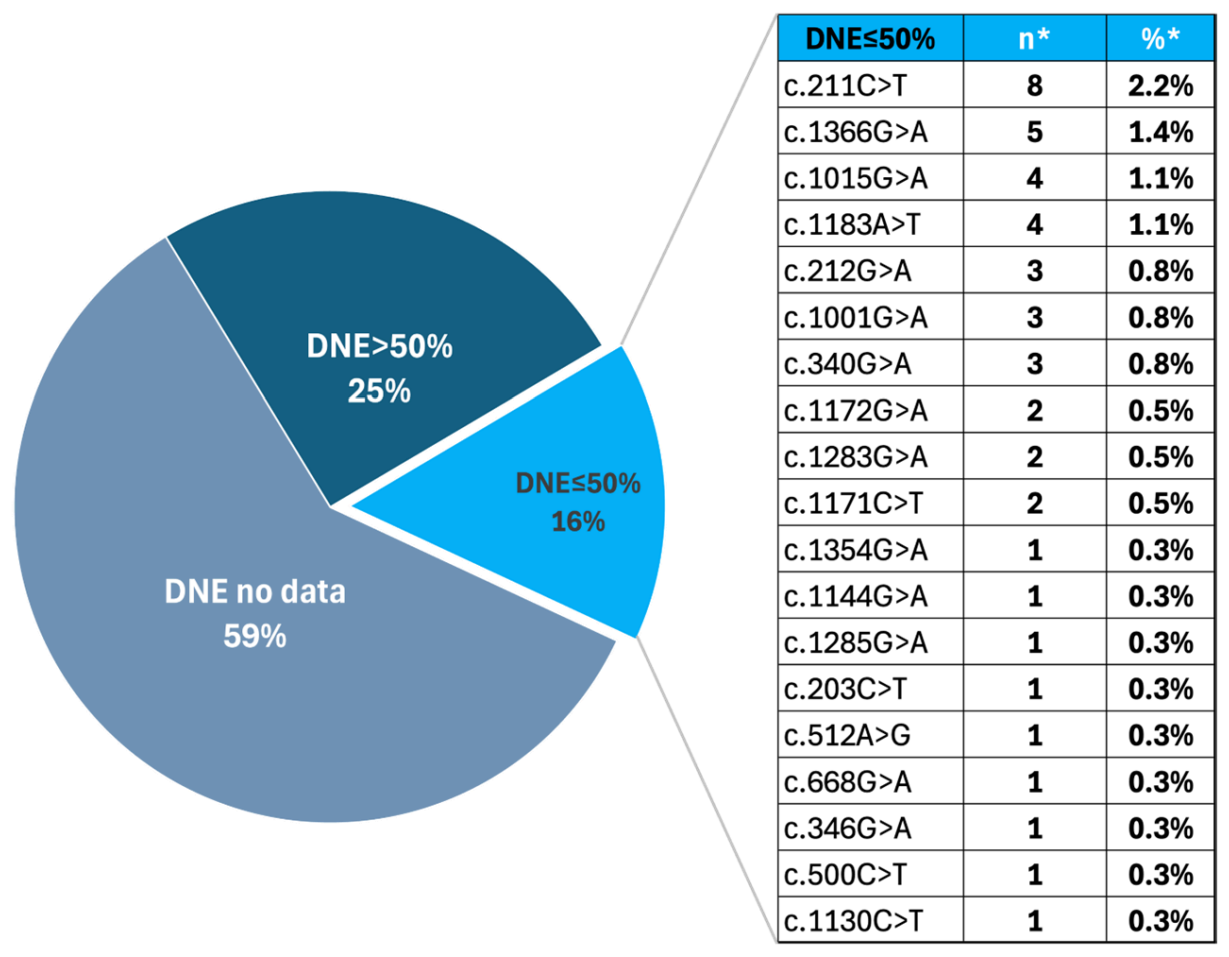
Dominant-negative effect. *—of the total number of identified nucleotide variants.

For 73 identified variants, data regarding dominant-negative effect (DNE) were unavailable. In 19 unique variants, the DNE was ≤50%. Among the variants with established DNE, the most frequent were c.211C>T-8 cases, c.1366G>A-5, c.1015G>A-4, and c.1183A>T -4 (Figure 4).

In a cohort of 3121 patients, 305 (9.8%) patients had variants in genes whose mutations can lead to diseases associated with impaired bone mineralization. A total of 303 variants in 32 genes were identified in the 305 patients, including 8 patients with biallelic causal variants in the *SERPINF1*, *SLC34A3*, *CREB3L1*, *P3H1*, *SNX10*, and *CYP2R1* genes, which lead to the development of diseases inherited in an autosomal recessive manner.

**Figure 5:**
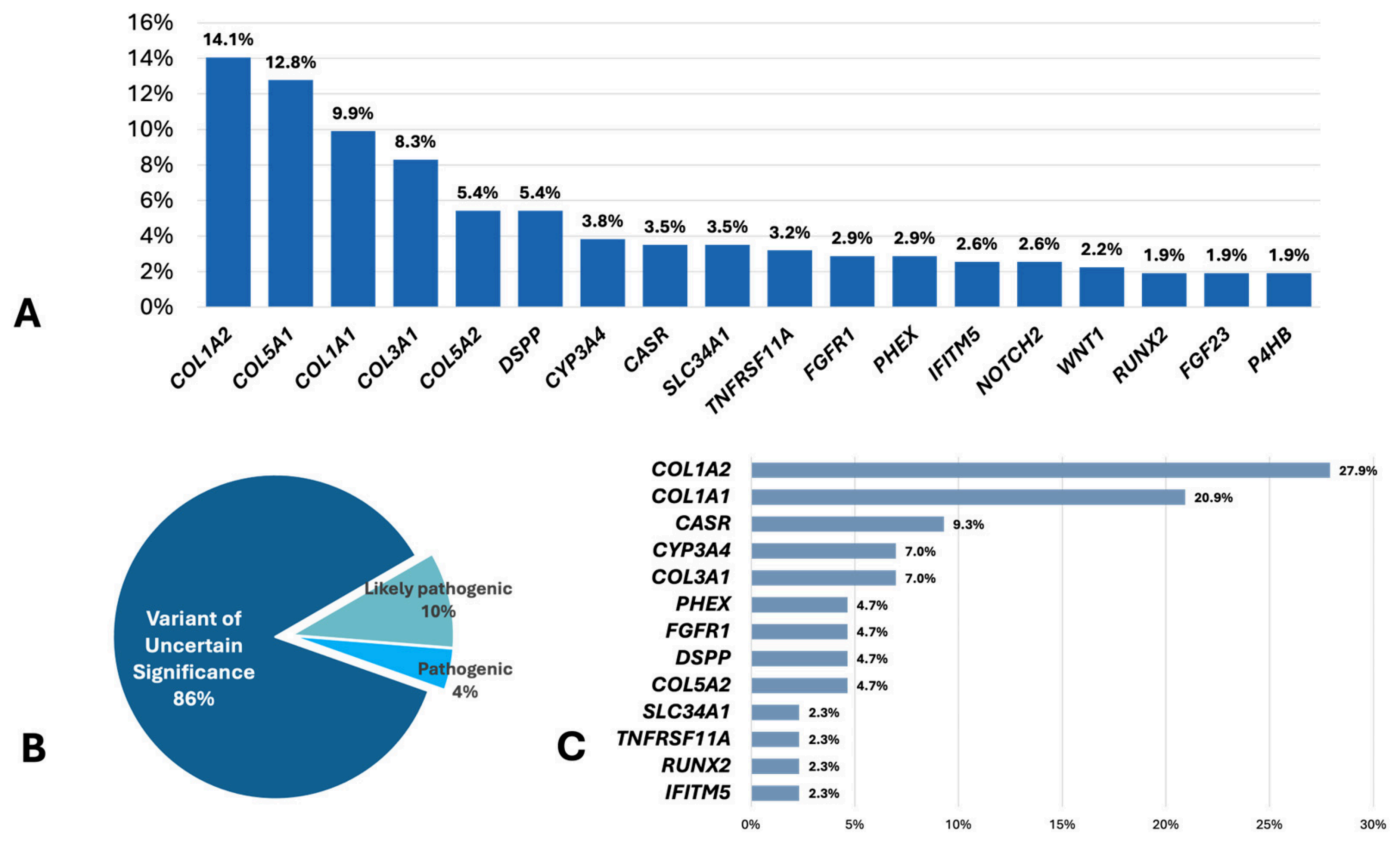
Characteristics of the pool of genetic variants identified by NGS. The panels display: non-*ALPL* genes with all types of variants (**A**), classification of findings by pathogenicity level (**B**), and the spectrum of loci with proven clinical significance (P/LP) (**C**).

The highest numbers of variants were identified in the: *COL1A2* genes—44 (14.1%); *COL5A1*—40 (12.8%); *COL1A1*—31 (9.9%); *COL3A1*—26 (8.3%); and *COL5A2* and *DSPP*—17 (5.4%) (Figure 5A).

Among the 313 causal variants, 13 (4.2%) were pathogenic, 30 (9.6%) were likely pathogenic, and the majority (270 (86.3%)) were classified as variants of unknown clinical significance (Figure 5B). Moreover, pathogenic and likely pathogenic causal variants were most frequently found in three genes: *COL1A2*-12 (27.9%), *COL1A1*-9 (20.9%) and *CASR*-4 (9.3%) (Figure 5C).

## 3. Discussion

In the case of severe HPP forms, timely differential diagnosis is crucial, especially given the availability of pathogenetic therapy. Monogenic disorders often allow for a definitive laboratory diagnosis through biochemical methods, such as measuring enzyme activity, followed by molecular genetic testing. Previously, we successfully applied these diagnostic algorithms using either Sanger sequencing [19] or NGS techniques with relatively small gene panels [20,21]. However, conditions with a complex phenotype, such as HPP, may require a more comprehensive approach.

There are no standardized “diagnostic pathways” for HPP. Newborn screening (NS) is instrumental for early detection of HPP, but currently HPP is not included in nation-wide NS anywhere in the world. Therefore, many research and clinical teams are currently working to develop optimal and cost-efficient strategy for timely differential diagnosis of HPP, combining clinical, biochemical, radiological assessments and genetic analysis, including but not limited to analysis of *ALPL* gene.

Selective screening for HPP among individuals with clinical characteristics suggestive of HPP was performed in the USA, with results published by Rush et al. in 2025 [22]. The cohort included 1113 individuals (775 adults and 338 children) who underwent Sanger sequencing of the *ALPL* gene. The most frequently identified P/LPVs were c.1133A>T (p.Asp378Val) (*n* = 61), c.571G>A (p.Glu191Lys) (*n* = 47), and c.1250A>G (p.Asn417Ser) (*n* = 44). P/LP NVs in *ALPL* were detected in 40% of participants, while, alarmingly, more than 50% had no identified variants despite reduced ALP activity and clinical symptoms typical of HPP. Was it because of the Sanger sequencing method limitations (suggesting that some of these patients had HPP, but the screening failed to establish correct diagnosis in their case)? Was it because NVs in other genes contributed to phenotype typical of HPP? To answer these questions deep NGS, NGS of large targeted gene panels, and whole exome sequencing or even whole genome sequencing (WES, WGS) should have been performed.

In our cohort, the most frequently found variants only partially overlapped with ones found by Rush et al. [22]; in particular, c.571G>A was also among frequently found ones (it was the most frequent in our cohort), but c.1133A>T and c.1250A>G were not found.

In Kumamoto Prefecture (Japan), from February 2019 to March 2022, a screening for HPP was conducted involving 45,632 newborns. The screening was performed using dried blood spots collected within the first week after birth, where TNSALP activity was measured. For newborns whose TNSALP activity remained below the cutoff in two subsequent tests, *ALPL* gene NGS was performed. Five newborns were found to carry heterozygous causal NVs in the *ALPL* gene: c.1304A>G (p.Asp435Gly), c.979T>C (p.Phe327Leu), c.734C>T (p.Thr245Met), c.1559delT (p.Leu520Argfs*86*), and c.1144G>A (p.Val382Ile). Among these variants was the one most common in Japan in patients with HPP (c.1559delT, p.Leu520Argfs*86*). The authors do not exclude the possibility that some children, whose TNSALP activity was above the threshold, or above the threshold upon re-testing, could be heterozygous carriers of causal *ALPL* gene variants. That study is the world’s first newborn screening for HPP. However, it did not cover the entire country but was limited to a single prefecture [23].

Given the differences between Russian and Japanese populations, it is not surprising that in our study c.1304A>G and c.979T>C were found only in one patient each (0.3%), and others most common in Noda’s study were not found.

In 2023, results were published from a prospective pilot study conducted in China which evaluated the potential of newborn genomic testing as a nationwide first-line screening method aimed at detecting rare genetic diseases. The cohort included 29,601 newborns from several screening centers across China. A large genomic panel consisting of 142 genes for 128 genetically determined diseases was developed, and targeted NGS was performed. Among the newborns screened, one asymptomatic infant with HPP was identified [24].

Despite the fact that traditional genetic analysis for diagnosis of HPP mostly relied on Sanger sequencing of *ALPL* gene, there is a strong tendency to use NGS-based approaches utilizing large panels of genes, combined with biochemical analysis including but not limited to measurement of ALP activity and levels of certain TNASP substrates. Our previously published work, conducted over a period from 2014 to 2021, has focused on the clinical and genetic characterization of pediatric patients whose clinical presentation suggests HPP, elucidating the frequency of HPP and spectrum of causative NVs in *ALPL* gene in the Russian population. The study utilized Sanger sequencing of *ALPL* gene in a large cohort of 1612 patients with reduced levels of ALP activity and identified 225 patients with heterozygous NVs in *ALPL* [25]. The most common P NV in *ALPL* was c.571G>A (p.Glu191Lys) (as well as in our current study), the second most common variant in the study by Glotov et al. was c.1171del (Arg391Valfs*12), which was found in our work only on 11 alleles (3%) [25].

A study by Kishnani et al. demonstrated a relationship between the localization of amino acid substitutions in the three-dimensional structure of TNSALP, and DNE of the corresponding NVs, as well as the enzymatic activity of TNSALP [26]. Allelic variants for which their causal roles in HPP and DNE have currently been confirmed are usually localized in the *ALPL* gene regions encoding one of the three domains of TNSALP: the homodimer-binding region, the active site, and the crown domain. This suggests that a variant located outside these regions likely does not lead to DNE. For example, the aforementioned missense variant c.571G>A causes clinical manifestations according to an autosomal recessive mode of inheritance; in vitro studies have shown a residual enzyme activity of approximately 68% and the absence of DNE in the case of this variant. Further, c.571G>A is often associated with mild forms of HPP, and in the study by Hérasse et al. it was found in 31% of patients with such clinical forms [27].

The most common P variant in *ALPL* in our cohort was also c.571G>A. This variant is very common in North Europe, supposedly resulting from a single mutation event. It is also found in North America and Australia, but is relatively rare in South Europe, and particularly frequent in Finland, reaching an allele frequency of 1/60 [12]. Apart from c.571G>A, according to Mornet et al. [12], a founder effect might be suggested for several other NVs due to their higher frequencies in some regions. For example, c.648 + 1 G>A, c.407G>A p.(R136H), and c.551G>A p.(R184Q) are particularly common in France; c.400_401delinsCA p.(T134H) in UK; c.1001G>A p.(G334D) and c.119C>T p.(A40V) in Germany; c.1015G>A p.(G339R) in North Europe; c.542C>T p.(S181L) in South Europe; c.1133A>T p.(D378V) in USA; and c.1250A>G p.(N417S) in UK and France. Of them, in our study cohort, c.1015G>A, known to be relatively common in North Europe, was also relatively frequent and found in 4 patients (1.37% frequency).

In total, 37 unique variants were identified in the *ALPL* gene in our study, namely c.613G>T, c.1189+2T>C, c.312T>G, c.1241T>C, c.1190-2_1190-1delinsGA, c.1055C>T, c.676A>G, c.329_330insCGCCGGCACCGCCAC, c.1437C>A, c.1237A>G, c.266T>C, c.325G>T, c.1313A>C, c.95A>G, c.661G>A, c.1229T>G, c.347C>T, c.911T>C, c.926T>G, c.419A>G, c.1342_1377del, c.966_973dup, c.1243_1244delinsCT, c.1314C>G, c.158del, c.366G>C, c.179A>G, c.1342C>A, c.1182dup, c.439G>A, c.1529C>G, c.182-5T>C, c.298-3C>T, c.-157C>G, c.648+5G>A, c.648+9delinsCC, and c.469G>A. These variants had not been previously described in the available literature or specialized databases, including LitVar, Varaico, LOVD, and JYU. Further systematization of the information about these variants is needed, including subsequent assessment of their clinical significance and functional impact on the protein.

Of particular importance in HPP is the identification of *ALPL* gene variants with a dominant-negative effect (DNE), in which the “mutant” protein disrupts the function of normal, wild-type TNSALP, leading to reduced enzymatic activity in heterozygous carriers. The presence of DNE is established when the residual enzymatic activity falls significantly below the 50% threshold in co-expression experiments, thereby differentiating this phenomenon from classical haploinsufficiency (loss-of-function).

Determining the presence of a DNE has significant practical importance not only for assessing the pathogenicity of identified variants and predicting disease course, but also for selecting therapeutic strategies [28]. In our study, we found 19 (16%) causal variants with previously confirmed DNE, verified in other studies.

An analysis of the percentage of patients with causal *ALPL* variants identified during various stages of the screening program revealed several key findings. The lowest detection rate of 3.3% in the Moscow cohort was recorded between September 2023 and February 2024, when, in addition to an ALP level below 150 U/L, the selection criteria for the screening predominately included nonspecific neurological and skeletal disorders. During the second stage (February–August 2024), when the patient profile of neurological and skeletal manifestations in selection criteria was somewhat narrowed, the detection rate increased to 5%. The highest percentage of detection rate, 12.2%, was reached at the third, current stage of the screening program (August 2024–March 2026).

It should be clarified that this stage consisted of two subprograms with virtually identical selection criteria, both of which identified an ALP level below the age- and sex-appropriate norm. In the first subprogram, the percentage of patients with causal *ALPL* variants was 8.8% (63/720), while in the second, it was significantly higher: 14.6% (182/1243). We carefully analyzed the enrolment criteria for selection and found that in the case of the second subprogram, selection for which occurred mainly among patients from outpatient appointments, one of the mandatory inclusion criteria was confirmation of a reduced level of ALP, the measurement was performed by colorimetric method using Roche Cobas analyzers. Thus, we can conclude that laboratory parameters were diagnostically significant in the selection criteria. The percentage of identified patients in the first subprogram was half as low, which may indicate less effective selection criteria and the inclusion of patients with a false decrease in the ALP levels. This could be caused by various factors, including preanalytical issues with the sample.

The low percentage (3.6%) of patients identified in the Saint Petersburg cohort is most likely due to the fact that many patients in this cohort were genotyped between 2022 and February 2024, when the selection criteria were less specific.

Given the variability of the symptoms of HPP, it can have a genetic complexity “beyond the *ALPL* gene”, and the clinical picture of the disease might be influenced by sequence features of other genes involved in bone and mineral metabolism or other HPP-related metabolic and signaling pathways. Some patients with NVs in *ALPL* also carried NVs (all of them being VUS) in one or more other candidate genes from the gene panel (Supplementary Table S1) [29,30], which, via an oligogenic mechanism, could have contributed to the disease presentation. Evaluating the combined effect of variants in multiple candidate genes may help clarify genotype–phenotype correlations and differences in clinical severity of HPP; however, this was beyond the scope of the current study. Notably, a recent case report presents an example of the patient subsequently co-diagnosed with osteogenesis imperfecta and HPP [31]. The therapy was adjusted based on the findings of genetic tests revealing the presence of the NVs in both *COL1A1* and *ALPL*, which once again highlights the importance of comprehensive genetic analysis for HPP therapy guidance.

Apart from *ALPL*, the commonly found in our cohort were NVs in genes *COL1A1* (encoding pro-alpha1(I) chain of collagen type I) and *COL1A2* (encoding pro-alpha2(I) chain of collagen type II), identified by NGS of the targeted panel of genes. The pathogenic sequence alterations in the *COL1A2* gene are primarily linked to osteogenesis imperfecta (OI), a rare genetic disorder characterized by “brittle bones”, caused by pathological changes in the structure and levels of type I collagen [32]. Thus, some clinical features of HPP and OI, such as skeletal abnormalities, overlap. Notably, a recent study found that individuals with deletions in the *COL1A2* gene also showed decreased expression of *ALPL* gene [33]. Our data showing that substantial numbers of patients suspected of HPP may carry P/LP NVs in genes *COL1A2* or *COL1A1* reinforces the view that analysis of these genes is useful to distinguish between OI and HPP.

In selective screenings for HPP among patients with reduced ALP activity, pathogenic variants in the *COL1A1* and *COL1A2* genes associated with osteogenesis imperfecta are frequently identified. It has been reported that osteogenesis imperfecta type II and campomelic dysplasia are the main differential diagnoses for HPP in utero, and osteogenesis imperfecta remains the most common differential diagnosis in infancy and childhood (typically type III and IV) [34]. Therefore, it is of importance to raise clinician awareness of overlapping phenotypes between osteogenesis imperfecta and HPP, such as multiple fractures with minimal trauma, long bone deformities, short stature, diffuse skeletal hypomineralization, bone pain, and muscle hypotonia (Supplementary Table S1). In our study, supporting information was provided to the specialist who referred the patient, including the list of genes included in the panel and conditions with symptoms overlapping with HPP. Furthermore, such report provided all the necessary information about the identified causal variants in the *ALPL* gene, as well as in the genes relevant for differential diagnosis.

Although most classical forms of osteogenesis imperfecta are characterized by normal or elevated ALP levels, severe variants may present with reduced enzyme activity due to marked osteoblast dysfunction and impaired bone matrix formation [35]. Accumulation of abnormal type I collagen within the endoplasmic reticulum of osteoblasts induces ER stress, disrupts cellular differentiation and osteoblast function, and may reduce the expression of tissue-nonspecific alkaline phosphatase. An additional contribution to decreased ALP levels is impaired interaction between osteoblasts and the defective extracellular matrix, along with altered differentiation of mesenchymal cells, which may result in biochemical characteristics resembling HPP [36]. The overlap between clinical and laboratory features of osteogenesis imperfecta and HPP has also been demonstrated in studies of patients presenting with low ALP, multiple fractures, and pathogenic variants in *COL1A1*/*COL1A2* genes [37]. Beyond the *COL1A1* and *COL1A2* genes, NVs in the following genes were also commonly identified by NGS of the targeted panel in NMRCCH cohort of patients with symptoms suggesting HPP: *CASR*, *CYP3A4* and *COL3A1*.

The *CASR* gene encodes G-protein-coupled calcium-sensing receptor (CaSR). The main function of CaSR is maintenance of extracellular Ca^2+^ homeostasis via regulation of parathyroid hormone (PTH) secretion and renal Ca^2+^ reabsorption/excretion [38]. Pathogenic NVs in the *CASR* gene are linked to disorders of calcium homeostasis such as familial hypocalciuric hypercalcemia (FHH) and autosomal dominant hypocalcemia (ADH) [39]. Of note, symptoms such as seizures, nephrocalcinosis, and hypercalcemia/hypercalciuria can occur both in cases of PNVs in the *CASR* gene and in severe forms of HPP. This is due to impaired mineral metabolism; however, the underlying mechanisms differ. In patients with PNVs in the *CASR* gene, the ALP activity level is typically within the normal range [40].

The *CYP3A4* gene encodes enzyme from cytochrome P450 family. P450 metabolizes endogenous and exogenous molecules, including metabolites of vitamin Da vitamin crucial for bone mineralization [41]. Bone disorders such as osteoporosis and osteomalacia are known to be related to vitamin D deficiency [42]. Thus, sequence alterations in *CYP3A4* gene may modulate metabolism of vitamin D and indirectly impact bone disorders. In vitamin D-dependent rickets type 3, patients may have symptoms common to HPP, such as rachitic bone changes, skeletal deformities, short stature, and muscle weakness. However, in these patients, ALP levels are typically elevated, which reflects a response to impaired mineralization [43].

The *COL3A1* gene encodes the pro-alpha chain of type III collagen, a fibrillar collagen that is found in extensible connective tissues such as skin, lung, uterus, intestine and the vascular system. PNVs in the *COL3A1* gene cause the vascular type of Ehlers–Danlos syndrome (OMIM 130050). This condition may present with symptoms overlapping with HPP, such as nonspecific musculoskeletal pain, weakness, and fractures. No changes in ALP levels have been reported in patients with causative *COL3A1* gene variants [44].

It is important to note that persistently reduced levels of TNSALP activity can also be observed in individuals without any pathogenic variants in the *ALPL* gene, highlighting the potential contribution of other genetic factors to TNSALP activity. For example, in a study by del Angel et al. published in 2025 [45], based on an analysis of genetic and biochemical data from individuals whose samples and data were biobanked in the UK Biobank, 12 genes influencing TNSALP activity were identified. In particular, variants in the *GPLD1*, *IFITM5*, *APOB*, *PCK1*, and *HSPG2* genes were associated with a decrease in the average TNSALP level [45]. Perhaps functional mRNA analysis might be needed to detect presence of splicing defects caused by such NVs, if deep sequencing (including whole genome sequencing, WGS) is not feasible to perform, but the phenotype of the patient clearly suggests HPP.

In this study, we present the results of a large-scale country-wide NGS-based selective screening for HPP among high-risk patients in Russia, providing a most comprehensive overview of HPP genetics landscape in Russian population to date. Notably, a large proportion of causal variants were identified in the genes from the differential diagnosis gene panel, highlighting the clinical utility of this approach for diagnosis of monogenic disorders associated with impaired bone mineralization. Nearly 10% of referred patients received a definitive molecular diagnosis, thereby ending their long diagnostic Odyssey. These findings support the potential of NGS as a first-tier testing strategy in future screening programs, including NS. Overall, using targeted NGS enhances our understanding of the genetic landscape of pediatric bone disorders including HPP, leading to informed decision-making when choosing tailored therapy strategies.

## 4. Materials and Methods

### 4.1. Patient Cohorts

The selection criteria were as follows.

In the 2022 selective screening program, patients with a confirmed ALP level below 100 U/L were included if they exhibited clinical features such as rickets-like skeletal changes or deformities, osteopenia or osteoporosis, premature loss of primary or permanent teeth, frequent or pathological fractures, delayed physical or motor development, musculoskeletal pain, osteomalacia, nephrocalcinosis or progressive renal disease, vitamin B6-dependent seizures, craniosynostosis, increased intracranial pressure, calcium pyrophosphate crystal deposition (pseudogout or chondrocalcinosis), joint stiffness, delayed walking, waddling gait, or respiratory insufficiency.

In 2023, the inclusion threshold was raised to an ALP level below 150 U/L while retaining the same clinical criteria.

From February 2024, the program focused on patients with ALP below 150 U/L who presented with a narrower set of features, including rachitic skeletal lesions, musculoskeletal pain, premature loss of primary teeth before the age of five, joint stiffness, pathological fractures, delayed walking with a waddling gait, delayed physical or motor development, or craniosynostosis.

From August 2024, the screening criteria were further refined and standardized, and patients with ALP activity below the age- and sex-adjusted reference range were considered eligible if they exhibited at least one of the following: skeletal or lower limb deformities, musculoskeletal pain, premature loss of primary teeth before age five, pathological fractures, craniosynostosis.

### 4.2. NGS

Genomic DNA was extracted from dried blood spots (DBS) and whole blood with the MagPure Universal DNA Kit (Magen, Guangzhou, China) using the automated Auto-Pure 96 DNA isolation station (Allsheng, Hangzhou, China). DNA quality and concentration were measured either by spectrophotometry with the NanoPhotometer N60 (Implen Gmbh, Munich, Germany), or with the Qubit dsDNA HS Assay Kit and Qubit 3.0 fluorometer (Invitrogen, Waltham, MA, USA).

The target regions of the *ALPL* and genes of differential diagnostics of HPP were analyzed. Hybridization probes for the NGS panel were created with the Hyper Design Tool (Roche Diagnostics, Indianapolis, IN, USA) to cover all coding and splice regions of the genes of differential diagnostics of HPP and whole sequence of the *ALPL*, including all exons and intronic regions. Library preparation followed the KAPA HyperPlus Kit protocol, using a 14 min DNA fragmentation to yield ~500 bp fragments. Target enrichment used KAPA HyperCap probes (Roche), and sequencing was performed on the MiSeq platform (Illumina Inc., San Diego, CA, USA) with V3 chemistry (600 cycles, paired-end). Each run generated an average of 24.5 million reads, 88% above Q30, with 99% of target regions covered at least 15 times and a mean read depth of 106×.

Bioinformatic analysis was performed according to the guidelines of Genome Analysis Toolkit Best Practices (https://gatk.broadinstitute.org, accessed on 6 July 2026). Briefly, raw reads were trimmed using Trimmomatic (version 0.39) and sequence alignment was performed with Burrows–Wheeler Aligner (version 0.7.17) using GRCh38 genome assembly as a reference. Next, duplicate reads were marked using Picard tools, and base quality score recalibration (BQSR) was performed. Then, genetic variations (SNPs and indels) were identified with GATK HaplotypeCaller (version 4.1.2). Next, gene annotation was performed with an in-house script to annotate variations present in ClinVar, OMIM, and HGMD databases.

All sequence variants with minor allele frequency of <1% according to the Genome Aggregation Database (gnomAD) (available at: https://gnomad.broadinstitute.org, accessed on 6 July 2026), were subjected to bioinformatics analysis in the program “Alamut Visual” (Interactive Biosoftware). In silico tools SIFT, PolyPhen HDIV, PolyPhen HVAR, Mutation Taster, FATHMM, CADD13, DANN, M-CAP and REVEL, as well as Alamut with built-in software modules, were used to predict the pathogenicity of the missense sequence variants. The guidelines of the American College of Medical Genetics and Genomics (ACMG) manual were used for variant interpretation [46].

In addition to the primary pipeline designed to detect single-nucleotide variants, we used a CNVkit-based script to identify copy number variations (CNV) in the *ALPL* gene. Three samples were flagged as potential CNV carriers by this approach; however, MLPA validation (MRC Holland P484 kit) showed that all these findings were false positives.

NGS panel 1 (Moscow cohort) included the full sequence of *ALPL* gene and 56 genes relevant to the differential diagnosis of disorders associated with impaired bone mineralization, namely *ALPL*, *ANKH*, *B4GALT7*, *BMP1*, *CASR*, *CLCN5*, *COL1A1*, *COL1A2*, *COL3A1*, *COL5A1*, *COL5A2*, *CREB3L1*, *CRTAP*, *CTNS*, *CYP27B1*, *CYP2R1*, *CYP3A4*, *DMP1*, *DSPP*, *ENPP1*, *FAM20C*, *FGF23*, *FGFR1*, *FKBP10*, *GALNT3*, *IFITM5*, *LIFR*, *MBTPS2*, *MGP*, *NHERF1*, *NOTCH2*, *OCRL*, *P3H1*, *P4HB*, *PHEX*, *PLOD2*, *PLS3*, *PPIB*, *PTDSS1*, *RUNX2*, *SEC24D*, *SERPINF1*, *SERPINH1*, *SGMS2*, *SLC34A1*, *SLC34A3*, *SLC39A13*, *SNX10*, *SOX9*, *SP7*, *SPARC*, *TMEM38B*, *TNFRSF11A*, *TNFRSF11B*, *VDR*, *WNT1*, and *XYLT2.* A detailed list of differential diagnoses conditions with clinical features similar to HPP and the associated causative genes is presented in Supplementary Table S2.

NGS Panel 2 (Saint Petersburg cohort) included the *ALPL* gene and 103 other genes associated with monogenic orphan diseases but did not include genes relevant to the differential diagnosis of HPP: *AKT3*, *NLRP3*, *TNFRSF1A*, *ALPL*, *TSC1*, *TSC2*, *TPP1*, *AGXT*, *LIPA*, *RPE65*, *NF1*, *MVK*, *NAGS*, *CPS1*, *OTC*, *ASS1*, *ASL*, *ARG1*, *SLC25A15*, *SLC25A13*, *SLC7A7*, *PHEX*, *MAN2B1*, *PAH*, *CTNS*, *PCCA*, *PCCB*, *IVD*, *MMUT*, *SCN1A*, *CBS*, *MTHFR*, *FGFR3*, *CFTR*, *IDUA*, *IDS*, *SGSH*, *NAGLU*, *HGSNAT*, *GALNS*, *ARSB*, *SERPINA1*, *PKD2*, *BRCA1*, *BRCA2*, *BTD*, *ATP7B*, *DMD*, *F8*, *F9*, *FAH*, *GAA*, *GALC*, *GALT*, *GBA*, *GCDH*, *GJB2*, *GLA*, *GLB1*, *HBB*, *HEXA*, *HEXB*, *NPC1*, *PKHD1*, *SLC26A2*, *HFE*, *PTPN11*, *DHCR7*, *CYP21A2*, *ABCD1*, *ACADM*, *ACADVL*, *ATM*, *BCKDHA*, *BCKDHB*, *DBT*, *L1CAM*, *PC*, *PPT1*, *SLC26A4*, *SLC6A19*, *USH2A*, *BLM*, *ASPA*, *IKBKAP*, *MTRR*, *NF2*, *SMPD1*, *APC*, *MUTYH*, *RLR*, *LDLRAP1*, *APOB*, *PCSK9*, *KCNQ1*, *COL4A5*, *ALDOB*, *HSD3B7*, *AKR1D1*, *VLCAD*, *LCHAD*, *CPTI*, *CPTII*, *TFPD*.

### 4.3. Sanger Sequencing

Sanger sequencing was used for segregation analysis in patients with variants of uncertain significance (VUS) in the *ALPL* gene. Primers were designed using Beacon Designer 8.10 (PREMIER Biosoft International, Palo Alto, CA, USA), and their specificity was verified with Primer-BLAST. Oligonucleotides were synthesized by JSC Evrogen. DNA was amplified on a Bio-Rad T100 thermocycler (Bio-Rad, Hercules, CA, USA). Sequencing reactions were performed with the BigDye^®^ Terminator v3.1 Cycle Sequencing Kit (Thermo Fisher Scientific, Waltham, MA, USA) according to the manufacturer’s protocol, followed by capillary electrophoresis on an ABI 3500XL Genetic Analyzer (Thermo Fisher Scientific, Waltham, MA, USA). The resulting sequences were compared with RefSeqGene reference sequences from the National Center for Biotechnology Information (NCBI) database.

## Data Availability

Data is unavailable due to privacy and ethical restrictions.

## Supplementary Materials

The following supporting information can be downloaded at: https://www.mdpi.com/article/10.3390/ijms27157008/s1.

## Abbreviations

The following abbreviations are used in this manuscript:

ACMG	American College of Medical Genetics and Genomics
AD	autosomal dominant
ALP	alkaline phosphatase
AR	autosomal recessive
BQSR	quality score recalibration
CNV	copy number variation
DNE	dominant-negative effect
ERT	enzyme replacement therapy
HPP	Hypophosphatasia
LP	likely pathogenic
NS	newborn screening
NGS	next-generation sequencing
NVs	nucleotide variants
P	Pathogenic
SNPs	Single-nucleotide polymorphisms
TNSALP	Tissue-nonspecific alkaline phosphatase
VUS	Variant of uncertain significance
